# Neural Network Differential Equations For Ion Channel Modelling

**DOI:** 10.3389/fphys.2021.708944

**Published:** 2021-08-04

**Authors:** Chon Lok Lei, Gary R. Mirams

**Affiliations:** ^1^Institute of Translational Medicine, Faculty of Health Sciences, University of Macau, Macau, China; ^2^Department of Biomedical Sciences, Faculty of Health Sciences, University of Macau, Macau, China; ^3^School of Mathematical Sciences, Faculty of Science and Engineering, University of Nottingham, Ningbo, China; ^4^Centre for Mathematical Medicine & Biology, School of Mathematical Sciences, University of Nottingham, Nottingham, United Kingdom

**Keywords:** neural networks, differential equations, electrophysiology, ion channels, mathematical modelling, model discrepancy, human Ether-à-go-go-Related Gene, neural ODEs

## Abstract

Mathematical models of cardiac ion channels have been widely used to study and predict the behaviour of ion currents. Typically models are built using biophysically-based mechanistic principles such as Hodgkin-Huxley or Markov state transitions. These models provide an abstract description of the underlying conformational changes of the ion channels. However, due to the abstracted conformation states and assumptions for the rates of transition between them, there are differences between the models and reality—termed model discrepancy or misspecification. In this paper, we demonstrate the feasibility of using a mechanistically-inspired neural network differential equation model, a hybrid non-parametric model, to model ion channel kinetics. We apply it to the hERG potassium ion channel as an example, with the aim of providing an alternative modelling approach that could alleviate certain limitations of the traditional approach. We compare and discuss multiple ways of using a neural network to approximate extra hidden states or alternative transition rates. In particular we assess their ability to learn the missing dynamics, and ask whether we can use these models to handle model discrepancy. Finally, we discuss the practicality and limitations of using neural networks and their potential applications.

## 1. Introduction

Electrophysiology modelling has provided insights insights into physiological mechanisms, from the ion channel to whole organ scales. Mathematical models of many ion channels, pumps, and exchangers form models describing the cellular action potential, based on the pioneering work of Hodgkin and Huxley (1952). These models of ion channels are typically a collection of mathematical functions governed by systems of ordinary differential equations (ODEs), using the Hodgkin-Huxley formulation or the Markov model structure (Rudy and Silva, 2006; Whittaker et al., 2020), and form the foundation of many cellular action potential, including neurons (Hodgkin and Huxley, 1952; Traub et al., 1994; Kole et al., 2008; Hay et al., 2011), cardiomyocytes (Noble, 1962; ten Tusscher et al., 2004; Grandi et al., 2011; O'Hara et al., 2011), pancreatic islet cells (Chay and Keizer, 1983; Sherman et al., 1988; Fridlyand et al., 2003; Cha et al., 2011), etc.

Both formulations of ion channel models provide an abstract description for the underlying conformational changes of the ion channels. The Hodgkin-Huxley formulation models the channels as independently-acting channel “gates” which can be open and closed. For example, a commonly used model for potassium ion channels is a combination of an activation gate and an inactivation gate. As the names imply, each of the gates attempts to describe a different behaviour that gives rise to the characteristic dynamics of the currents.

Many ion channels involved in generating action potentials are voltage-gated. A Hodgkin-Huxley gate for voltage-gated ion channels is usually modelled as

(1) $$\begin{array}{l} \text{closed }\overset{\alpha (V)}{\underset{\beta (V)}{⇌}}\text{open}, \end{array}$$

where α and β are the *transition rates* between the open and closed states, and *V* is the *membrane voltage*. Then the open probability of the gate, *x*, can be expressed as

(2) $$\begin{array}{l} \frac{\text{d}x}{\text{d}t}=f\left(x,V\right), \end{array}$$

(3) $$\begin{array}{l} f\left(x,V\right)=\alpha \left(V\right)\left(1-x\right)-\beta \left(V\right)x, \end{array}$$

(4) $$\begin{array}{l} \alpha \left(V\right)=A_{\alpha }\exp\left(B_{\alpha }V\right), \end{array}$$

(5) $$\begin{array}{l} \beta \left(V\right)=A_{\beta }\exp\left(B_{\beta }V\right), \end{array}$$

where *f*(*x, V*) represents a function for the rate at which gating occurs. In the case of Equation (1), mass-action kinetics dictate that *f*(*x, V*) takes the form shown in Equation (3) in terms of α(*V*) and β(*V*) (as introduced by Hodgkin and Huxley, 1952). A canonical form for α(*V*) and β(*V*) is shown in Equations (4, 5), so that {*A*_α_, *B*_α_, *A*_β_, *B*_β_} are the four constants/parameters governing this gate. The voltage-dependence shown in Equations (4, 5) is not always used for all rates in Hodgkin-Huxley models [indeed in all three of the gates in their original model Hodgkin and Huxley (1952) used this form for only one of the two rates, fitting the other empirically] but it has some biophysical justification in terms of Eyring transition rate theory to support the exponential form of the dependence on voltage (Lei et al., 2019a). Indeed it is more common to see Equations (4, 5) used for Markov model state transition rates, but we and others have found it works very well for Hodgkin-Huxley models for a range of currents (Lei et al., 2019a; Houston et al., 2020). One could also construct a model with multiple closed states to describe different dynamics (see section 2.6, and Rudy and Silva, 2006 for a review).

Often we find we have a more predictive model for some of the processes than the others. For example, for the rapid delayed rectifier current (I_Kr_) a simple Hodgkin-Huxley gate can describe the fast inactivation process better than the slower activation process (Beattie et al., 2018; Lei et al., 2019a,b). We might then wish to “correct” the model discrepancy of the slower activation process, but “trust” the mechanistic model for the faster inactivation process. We propose to use neural networks as a universal approximator to learn the dynamics of individual gating processes of ion channels. In such a case, we would then alter just part of the model (some of the equations).

Neural networks have a kind of universality which can be used to approximate any arbitrary (well-behaved) function (Cybenko, 1989; Leshno et al., 1993; Pinkus, 1999). One could attempt to learn the output (current) or the discrepancy of the output directly using such an approximator, similar to the modelling approach for the discrepancy term described in Kennedy and O'Hagan (2001). However, Lei et al. (2020c) investigated such an approach and discussed its limitations, and suggested the need for passing in the “history” of the simulation to the approximator to predict the next time points when modelling dynamical systems.

Recently, there has been a growing amount of research in data-driven approaches or equation-learning methods for (numerically) modelling dynamical systems. Some of which work by approximating derivatives of states from data and regressing on these variables (e.g., Wu and Xiu, 2019); whilst others combine machine learning methods, such as deep neural networks, with prior domain knowledge encoded in differential equations (Chen et al., 2018; Rackauckas et al., 2020). A similar approach has recently been applied to model a simple cardiac electrophysiology system for replacing numerical integration of partial differential equations (Ayed et al., 2019). Given the success of modelling the dynamics of ion channels using a relatively simple ODE system (for I_Kr_, e.g., Beattie et al., 2018; Lei et al., 2019a,b), it would make sense to approximate or improve the right-hand side of the already “useful” ODE instead of trying to learn all the already well-captured biophysics from scratch. Such an approach is sometimes referred to as a *neural ODE* (Chen et al., 2018; Bonnaffé et al., 2021) or *ODE-Net* (Zhong et al., 2020). Note that we refer to “neural ODEs” as leveraging neural network terms within ODEs, which is different to some of the classification applications described in Chen et al. (2018) but similar to their ODE applications.

In this paper, we use the *human Ether-à-go-go-Related Gene* (hERG) potassium ion channel, which carries the cardiac current I_Kr_ (Sanguinetti et al., 1995), as a working example to demonstrate the feasibility and practicality of using neural ODEs to model ion channel kinetics. We provide an alternative modelling approach that could alleviate certain restrictions, such as the exponential form of the transition rates and the linear relationship of the states in Equation (3). We compare and discuss multiple ways of using a neural network to approximate the hidden states, the dynamics of hERG. Their ability to handle model discrepancy is assessed through synthetic data studies. We also apply variants of neural ODEs to real experimental data. Finally, we discuss the practicality of this approach and its potential applications.

## 2. Materials and Methods

We first introduce a Hodgkin-Huxley ion channel model which we adopt as our case study for this article. We then present the neural network modifications to the mechanistic ODE models, and methods to train the neural network models. Finally we describe synthetic data studies that we performed, and an application to real experimental data.

### 2.1. A Hodgkin-Huxley Ion Channel Model

We used a simple Hodgkin and Huxley-style hERG model as the working model (as used in Beattie et al., 2018). In this model, the current is modelled with a standard Ohmic expression,

(6) $$\begin{array}{l} I=g\cdot a\cdot r\cdot \left(V-E\right), \end{array}$$

where *g* is the maximal conductance, *a* is a Hodgkin-Huxley-style activation gate, and *r* is an inactivation gate. Both of these gates have transition rates following the form shown in Equations (2–5). *E* is the reversal potential for this potassium ion current, also known as the Nernst potential, which is not inferred but is calculated directly using

(7) $$\begin{array}{l} E=\frac{RT}{zF}\ln\;\left(\frac{{\left[{\text{K}}^{+}\right]}_{\text{o}}}{{\left[{\text{K}}^{+}\right]}_{\text{i}}}\right), \end{array}$$

where *R* is the ideal gas constant, *T* is the absolute temperature (*T*= 294.55 K for the data we use later), *F* is the Faraday constant, and *z* is the valency of the ions (equal to 1 for potassium ions). ${\left[{\text{K}}^{+}\right]}_{\text{o}}$ and ${\left[{\text{K}}^{+}\right]}_{\text{i}}$ denote the extracellular and intracellular concentrations of potassium ions, respectively, which are determined by the experimental solutions (${\left[{\text{K}}^{+}\right]}_{\text{o}}=$4 mM and ${\left[{\text{K}}^{+}\right]}_{\text{i}}=$ 110 mM in the data we use later). The two gates are (independently) modelled using Equation (3), giving a total of 8 parameters, each of which is to be determined from the experimental current recordings.

For hERG, the dynamics of inactivation (*r* gate kinetics) happen on a time scale much faster than the activation (*a* gate). A typical time scale of interest for action potential modelling is tens to hundreds of milliseconds. As a result, we observe more obvious errors in the dynamics of the *a* gate, provided the steady state of *r* is sufficiently accurate. In the rest of this paper, we assume the *r* gate equation and parameters after fitting to the data is accurate and we correct only the dynamics of the activation—the *a* gate—using the methods described in the next section.

### 2.2. Ion Channel Model With Neural Networks

To relax the assumption of the linearity of the gate variable relationship and the exponential rate constants we trialled modelling the entire gating dynamics using a neural network, replacing Equation (2) with:

(8) $$\begin{array}{l} \frac{\text{d}x}{\text{d}t}=N\left(V,x\right), \end{array}$$

where N(*V, x*) denotes a neural network that takes the voltage *V* and the state *x* as inputs (see next section for more details). This is perhaps the most flexible way to describe a Hodgkin-Huxley gate, as we allow a neural network to *fully* approximate the right-hand side of a gate's ODE (which we will call “*NN-full*” or “*NN-f* ”).

Instead of replacing the whole right-hand side of the ODE with a neural network, we also trialled using a neural network to model the *discrepancy* (“*NN-discrepancy*” or “*NN-d*”) between the ordinary mechanistic model *f* and the data generating process (or the true system), replacing Equation (2) with:

(9) $$\begin{array}{l} \frac{\text{d}x}{\text{d}t}=f\left(x,V\right)+N\left(V,x\right). \end{array}$$

In this case *f*(*x, V*) = α(1 − *x*) − β*x* as in Equation (3), but it could represent any other candidate model of the gate. The NN-d approach is similar to the “augment incomplete physical models for identifying and forecasting complex dynamics” framework proposed by Yin et al. (2020). In theory, given the flexibility of the neural network, as a universal approximator, Equation (9) should be able to provide a similar approximation as Equation (8).

The first approach is a purely data-driven neural ODE, where the entire dynamics are described by the neural network, making good use of their universal approximator property. The second approach utilises prior knowledge of the biophysics of the gating process, which perhaps gives us a good initial guess of the neural network should be around zero, treating the neural network as a model discrepancy term.

### 2.3. Neural Networks

We used a feedforward neural network, a multi-layer perceptron model (Goodfellow et al., 2016), to approximate the dynamics (hidden states) and/or to correct its discrepancy. A feedforward neural network defines a (nonlinear) map of an input vector to an output vector. Let N be an operator for a feedforward neural network with *M* hidden layers, such that it has *p* inputs and *q* outputs (ℝ^*p*^ → ℝ^*q*^). Given the inputs $x={\left[x_{1},x_{2},\ldots ,x_{p}\right]}^{T}\in {\mathbb{R}}^{p}$, the weights ***W***_*m*_ between the *m*^*th*^ and the (*m* + 1)^*th*^ layers, and the activation functions *h_m_* : ℝ → ℝ for each “neuron” or “node” in the *m*^*th*^ layer, the feedforward neural network computes the outputs $y={\left[y_{1},y_{2},\ldots ,y_{q}\right]}^{T}\in {\mathbb{R}}^{q}$. The mapping can be expressed as

(10) $$\begin{array}{l} y=N\left(x;\Theta \right)=W_{M+1}○\left(h_{M}○W_{M}\right)○\cdots ○\left(h_{1}○W_{1}\right)\left(x\right), \end{array}$$

where **Θ** is the parameters of the network weights, and ° denotes operator composition. The weight matrices include the network biases; the activation functions are applied in a component-wise manner.

For the models specified in section 2.2, the inputs ***x*** were the membrane voltage *V* and the ODE state *x*. The output ***y*** was the derivative of the state d*x*/d*t* for Equation (8) or the discrepancy in the derivative when using the mechanistic model *f* for Equation (9).

There are multiple ways of training such a neural network embedded within (part of) the right-hand side of the differential equation system. Su et al. (2021) suggested using pairs of consecutive time series data points as the training data for the neural networks; an alternative would be the adjoint method proposed by Chen et al. (2018), see section 4. The method proposed by Su et al. (2021) is equivalent to estimating the derivative of the data (without smoothing) using a first order forward finite difference scheme. Here we propose an alternative method that we term “state space estimation,” which can be used to train the neural network for learning the dynamics of the gating processes in a similar manner, as described in the next section.

### 2.4. State Space Estimation

In voltage-clamp experiments, we measure the current from the cell by holding the membrane voltage at various levels. The current model in Equation (6) can be generalised for any Hodgkin-Huxley current as

(11) $$\begin{array}{l} I=g\cdot \underset{k}{\prod }{\left(x_{k}\right)}^{n_{k}}\cdot \left(V-E\right), \end{array}$$

where *k* indexes the distinct gating variables *x*_*k*_, each of which is governed by its own ODE (Equation 2), and is raised to an integer power *n*_*k*_, and *g, E* are constants as discussed above. We assume that we are interested in estimating the state space of the gate *x*_*i*_, and that we can model the other gates *x*_!*i*_ (where the subscript !*i* represents all *k* except *i*) and can observe only the current *I* directly. Here for the models of interest, the state space of the gate *x*_*i*_ is its derivative d*x*_*i*_/d*t* as a function of *x*_*i*_ and *V* (see later Figure 2 that shows an example of the state space in the synthetic data studies).

There are two ways of estimating the state space of the gate *x*_*i*_. First, we can directly estimate the state by rearranging Equation (11) in terms of modelled/known quantities

(12) $$\begin{array}{l} x_{i}={\left(\frac{I}{g\cdot \underset{k\neq i}{\prod }x_{k}^{n_{k}}\cdot \left(V-E\right)}\right)}^{\frac{1}{n_{i}}}. \end{array}$$

Then we can approximate the derivative of Equation (12) by fitting either a spline or some differentiable closed-form expression (such as sums of exponential functions for fixed voltage levels), which gives us an estimate of d*x*_*i*_/d*t* as a function of *x*_*i*_ and *V* for *V* ≠ *E* and *x*_*k*_ ≠ 0 for all *k* ≠ *i*. However, the denominator of Equation (12) can get arbitrarily close to zero, which can amplify noise in the current *I*, causing very different noise levels at different regions of the signal for fitting.

Alternatively, to derive the derivative of *x*_*i*_, we assume we have models which will provide the numerical derivatives for all *x*_!*i*_; usually we have the analytical form of the derivatives for all *x*_!*i*_. We would also need to estimate the derivative for *I* numerically, for example by fitting a spline to *I* (usually we do not have simple differentiable closed-form expression for *I*); we used a univariate smoothing cubic spline provided by Python SciPy (Virtanen et al., 2020), and we fitted a separate spline on each discontinuous step in *V* to capture discontinuities in *I* as a result of a sudden change in the driving term (*V* − *E*) in Equation (6). An example of the spline fitting results is shown for the synthetic data studies. By applying product rule to Equation (11) we notice that the current derivative approximated by a spline is also equal to

(13) $$\begin{array}{l} \frac{\text{d}I}{\text{d}t}=g\cdot (V-E)\cdot (n_{i}x_{i}^{\left(n_{i}-1\right)}\frac{\text{d}x_{i}}{\text{d}t}\prod_{k\neq i}x_{k}^{n_{k}}\\ \;+x_{i}^{n_{i}}\sum_{j}n_{j}x_{j}^{\left(n_{j}-1\right)}\frac{\text{d}x_{j}}{\text{d}t}\prod_{k\neq i,j}x_{k}^{n_{k}})+g\prod_{k}x_{k}^{n_{k}}\frac{\text{d}V}{\text{d}t}, \end{array}$$

which can be rearranged to get an estimate for the derivative of the state of interest

(14) $$\begin{array}{l} {\frac{\text{d}x_{i}}{\text{d}t}|}_{\left(x_{i},V\right)}=\frac{1}{n_{i}x_{i}^{\left(n_{i}-1\right)}\prod_{k\neq i}x_{k}^{n_{k}}}(\frac{1}{\left(V-E\right)}\left(\frac{1}{g}\frac{\text{d}I}{\text{d}t}-\prod_{k}x_{k}^{n_{k}}\frac{\text{d}V}{\text{d}t}\right)\\ \;-\sum_{j}n_{j}x_{j}^{\left(n_{j}-1\right)}\frac{\text{d}x_{j}}{\text{d}t}\prod_{k\neq j}x_{k}^{n_{k}}). \end{array}$$

With Equations (14) and (12), we again obtain d*x*_*i*_/d*t* as a function of *x*_*i*_ and *V* for *V* ≠ *E* and *x*_*k*_ ≠ 0 for all *k* ≠ *i*.

These results of state space estimation can then be used as the training data for the neural networks in section 2.2. This method can also be useful to check either Equation (3) is a good approximation to the gating dynamics (e.g., if d*x*_*i*_/d*t* is linear in *x*_*i*_) or Equations (8) or (9) is needed to approximate the surface d*x*_*i*_/d*t*.

### 2.5. Data Preparation and Network Training

The raw time series data were processed by using the state-space estimation, giving a set of tuples (*a, V*, d*a*/d*t*). We normalised the data by a simple scaling normalisation by (1, 100, 1, 000) such that each variable in the tuples is O(1), which is commonly advised to preprocess neural network training data (Bishop et al., 1995). The loss function is defined as the mean squared error,

(15) $$\begin{array}{l} L_{f}\left(\Theta \right)=\frac{1}{T}\overset{T}{\underset{t=1}{\sum }}{\left(\frac{\text{d}a}{\text{d}t}{|}_{t}-N\left(x_{t},V_{t};\Theta \right)\right)}^{2}, \end{array}$$

for the NN-f model, where *T* is the number of data points; the loss function for the NN-d model is

(16) $$\begin{array}{l} L_{d}\left(\Theta \right)=\frac{1}{T}\overset{T}{\underset{t=1}{\sum }}{\left(\frac{\text{d}a}{\text{d}t}{|}_{t}-f\left(x_{t},V_{t}\right)-N\left(x_{t},V_{t};\Theta \right)\right)}^{2}, \end{array}$$

where *f* is the candidate model for the activation *a*-gate specified by Equation (3). By minimising the loss function, we obtained a set of trained neural network parameters Θ^*^. For any given new initial conditions or voltage clamp, we can use the trained model to perform predictions. The equations were solved using the Runge-Kutta of order five of Dormand-Prince-Shampine (dopri5) via the open source package torchdiffeq by Chen et al. (2018), with tolerance settings for the solver set to atol = 10^−6^ and rtol = 10^−8^. All codes and data are freely available at: https://github.com/chonlei/neural-ode-ion-channels.

For all the neural network models, we used a fully connected network with five hidden layers, each of which has 200 nodes, and with the leaky-rectified linear unit (ReLU) as the activation function to account for the nonlinearity between the inputs and outputs. The nodes in the input layer consisted of the scaled state variable *a* (activation gate) and the scaled control variable *V* (membrane voltage), and the output layer is the scaled derivative of the state variable d*a*/d*t*. Networks with different depth and width have been investigated; grid search across {1, 5, 10} layers and {10, 100, 200, 500} nodes were performed with the NN-f model for the real cell dataset and the results are shown in Supplementary Table 1. All neural network models were trained using Adam's algorithm (Kingma and Ba, 2017) via the open source PyTorch library (Paszke et al., 2019).

### 2.6. Synthetic Data Studies

**Figure 1:**
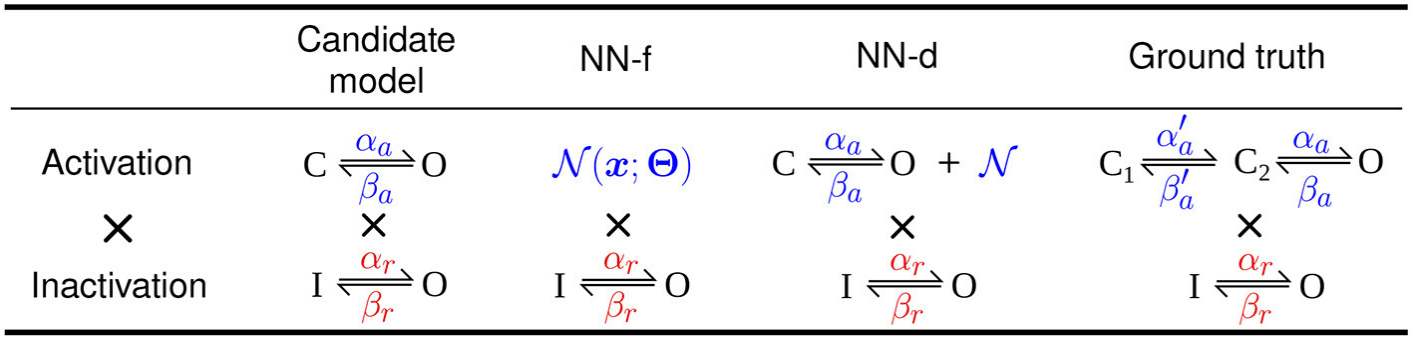
Models of hERG used in synthetic data studies. studies. From left to right shows the original Hodgkin-Huxley model (candidate model), the activation modelled using a neural network (NN-f), the activation with a neural network discrepancy term (NN-d), and the activation modelled with a three-state model (ground truth). All models have the same (independent) inactivation.

We performed synthetic data studies to assess whether the neural network, in the forms of NN-f and NN-d, can approximate the missing dynamics of the activation in the Hodgkin-Huxley model in Equation (6). We used a different model, a “ground truth” model, to generate the synthetic data, such that this synthetic data study inherently had discrepancy between the candidate model and the data; as well as using the ground truth model to generate data (with model discrepancy), we tested the approach using the candidate model (with no model discrepancy) and showed that the neural ODE models were fully capable of capturing the kinetics of the candidate model (see later of this section). We used a three-state Markov model for the activation to be the ground truth model for generating the synthetic data. The simpler two-state model of the activation was the candidate model, which cannot fully capture the dynamics of certain parameterisation of the ground truth model. This sets the challenge to use the methods in section 2.2 to capture the missing dynamics. Figure 1 shows the model structures of the two models (Markov representations of these two models are shown in Supplementary Figure 1) and schematics for the NN-f and NN-d models. Note that we do not necessarily believe one model is better than the other, as we noticed neither the candidate model nor the “ground truth” model could capture the full dynamics of real experimental hERG data.

**Figure 2:**
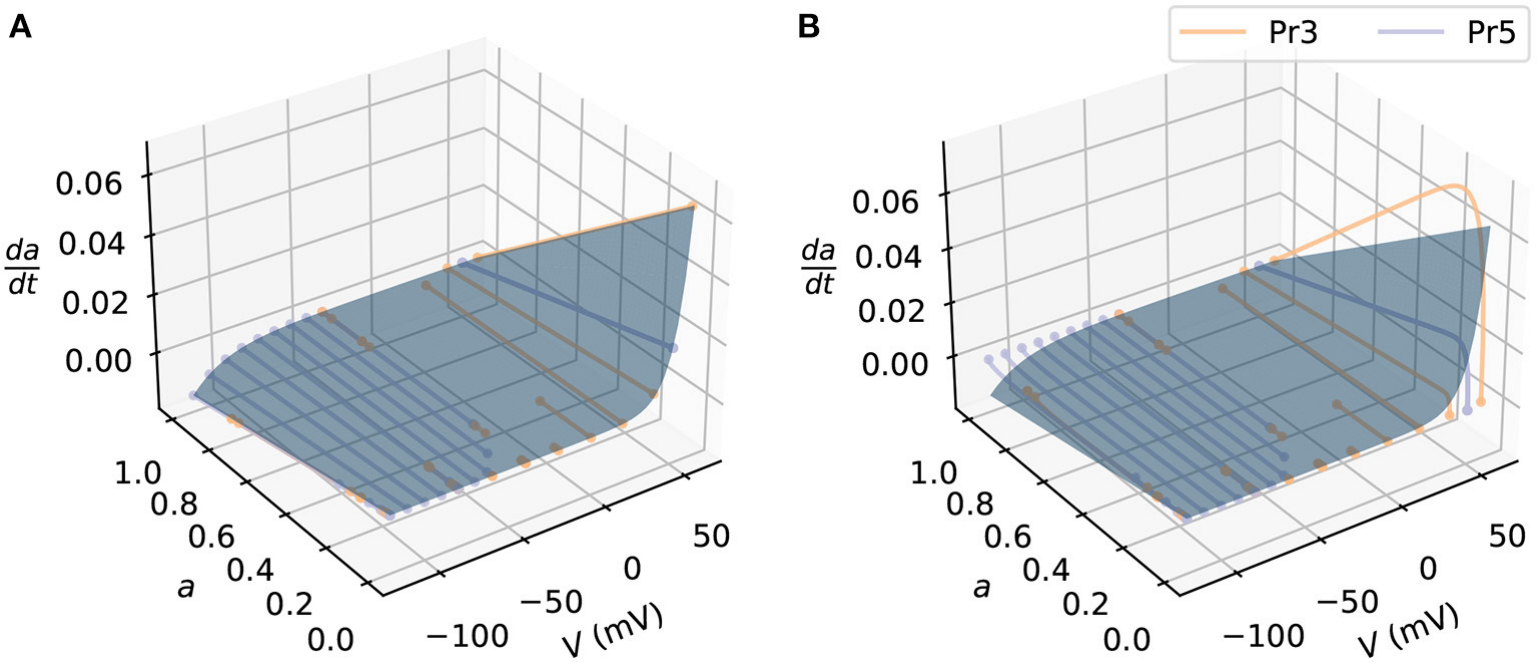
An example of the state space simulated in synthetic data studies. The state space of the candidate model (blue surface) is shown as blue surfaces. The simulated activation steady-state protocol (Pr3, orange lines) and the simulated deactivation time constant protocol (Pr5, purple lines) are shown for **(A)** the candidate model and **(B)** the ground truth model. Each dot at the two ends of the lines indicates a voltage step jump in the protocols.

We generated the synthetic data by simulating the current *I*, with some fixed known parameter sets, voltage protocol *V*(*t*), initial conditions, and sampling time (time-step). We used the kinetic parameters identified from a previous study (Lei et al., 2019b) in the synthetic data studies, as given in Supplementary Table 2, whilst setting the maximum conductance *g* to 1 μS. For the voltage protocol, we used an activation steady state protocol (Pr3) and a deactivation protocol (Pr5) from Beattie et al. (2018) for training the activation process of the models—the same protocols will later be used for the real data in section 2.7. Figure 2 shows the state space of the activation *a*-gate model covered by the training protocols. These protocols were designed to explore the dynamics for the activation process in hERG, making them an appropriate choice for training hERG activation kinetics; they were also able to elicit currents that allow identifiability for the candidate hERG model parameters (see e.g., “Method 3” in Clerx et al., 2019a). For the initial conditions, since the cells in the experiments in Beattie et al. (2018) were held at -80 mV prior to running the voltage protocols, we use the steady state values of -80 mV as the initial conditions; we also used the same sampling time points as the data. After simulating the outputs using the ground truth model, we added independent and identically distributed Gaussian noise (with zero nA mean and 0.1nA standard deviation) to the outputs, to generate the synthetic dataset.

We applied the state-space estimation methods to postprocess the noisy time series data for training the neural networks; Supplementary Figure 2 shows an example of the spline fitting results. Figure 2B shows the discrepancy in the state space between the candidate model and the ground truth model simulated with the training protocols that the neural network models will learn. The candidate model was fitted using a Python open source package PINTS (Clerx et al., 2019b), with the fitted parameters given in Supplementary Table 3. After training the models, we further assessed the model by predicting unseen protocols, including an inactivation time constant protocol (Pr4), the “sinusoidal” protocol (Pr7), and a collection of action potential wave forms (Pr6) that featured in Beattie et al. (2018). This check ensures the models learned the appropriate dynamics of the underlying system instead of simply overfitting (Whittaker et al., 2020).

To demonstrate the neural network models are fully capable of modelling the candidate model, we also repeated this synthetic data study with data generated from the candidate model (i.e., no discrepancy). The results are shown in Supplementary Figures 3, 4, showing both neural network models were able to fully capture the dynamics of the candidate model.

### 2.7. Application to Experimental Data

We applied the neural network differential equation models, NN-f and NN-d, to experimental data taken from Beattie et al. (2018, Cell #5). In brief, manual patch-clamp recordings were performed at room temperature (between 21 and 22°C) in Chinese hamster ovary (CHO) cells stably expressing hERG1a which encodes the α subunit of the channel carrying I_Kr_. The experiments consisted of seven protocols, Pr1–Pr7 with the numbering matching the original publication; see Beattie et al. (2018) for details on postprocessing experimental data. Following Beattie et al. (2018), capacitance artifacts were removed from the experimental data by discarding the first 5 ms after each discontinuous voltage step.

Similar to the synthetic data studies, we applied the state-space estimation methods to postprocess the time series data measured with the activation steady state protocol (Pr3) and the deactivation protocol (Pr5) for training the neural network models. The trained models were then used to predict unseen protocols: the inactivation time constant protocol (Pr4), the sinusoidal protocol (Pr7), and a series of action potential wave forms (Pr6).

## 3. Results

### 3.1. Neural Network ODEs Capture Missing Dynamics in Synthetic Data

**Figure 3:**
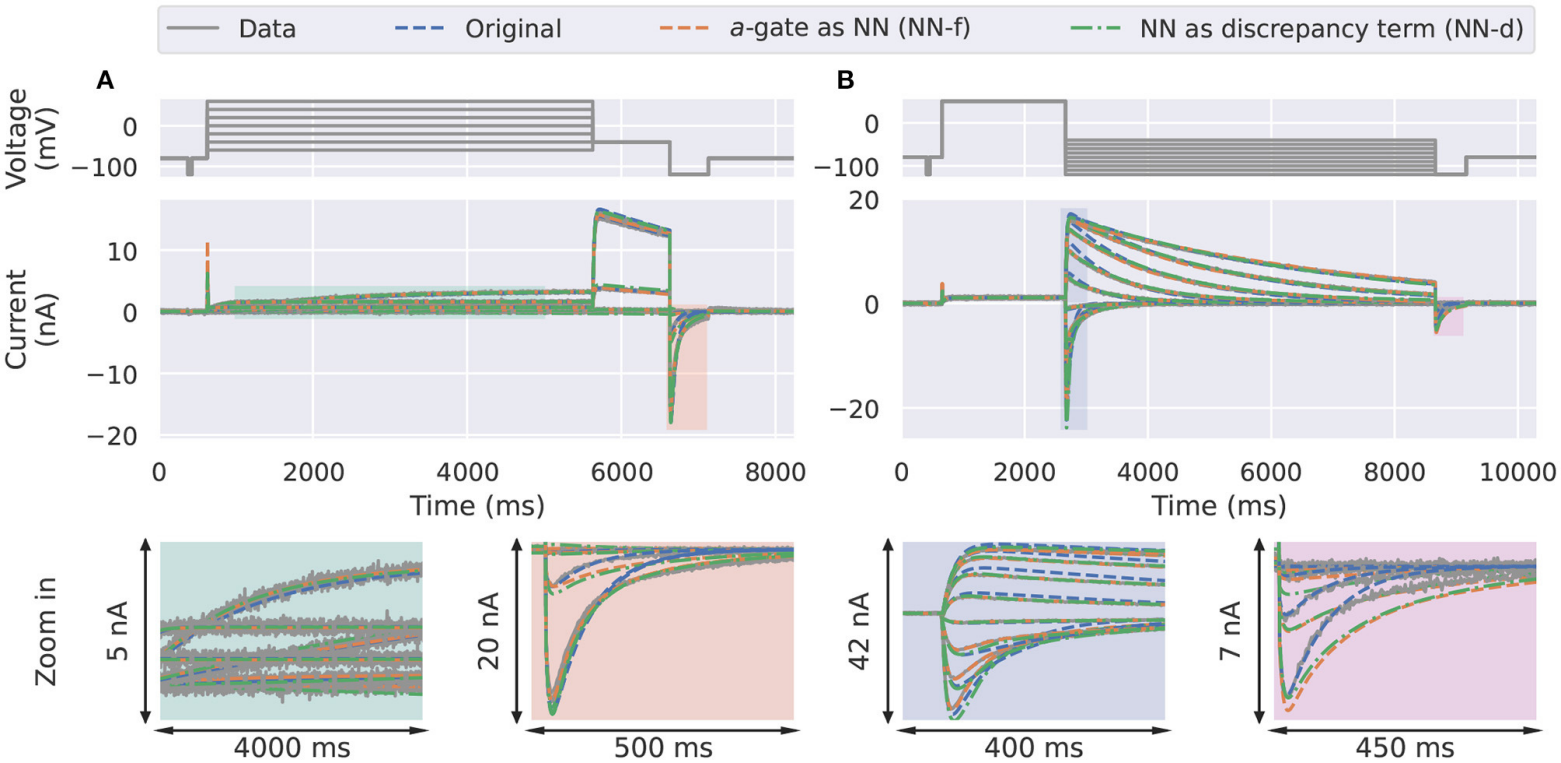
Training results for the synthetic data studies. The training data generated using the ground truth model (grey) are compared against the original candidate model (blue), the *a*-gate modelled using a neural network (NN-f, orange), and the *a*-gate with a neural network discrepancy term (NN-d, green). **(A)** Shows the activation steady-state protocol (Pr3) and **(B)** shows the deactivation time constant protocol (Pr5). The top panels show the voltage-clamp protocols, the middle panels show the currents, and the bottom panels show the magnification of part of the currents. All figures with a blue background are synthetic data examples.

In the synthetic data studies, we attempted to fit a standard Hodgkin-Huxley *a*-gate model (Equation 3, candidate model), the NN-f model (Equation 8), and the NN-d model (Equation 9) to the synthetic data, where the data were generated using a three-state activation model. The training results are shown in Figure 3, comparing the ability of the neural ODE models to learn the dynamic behaviour of the system under the training data sets: the activation steady-state protocol (Pr3) and the deactivation time constant protocol (Pr5). The candidate model (blue) was not able to fit to some of the “two time constant” dynamics at the end of the activation protocol (magnification shown in orange) and the beginning of the deactivation protocol (magnification shown in blue).

The NN-f model (orange), where the entire *a*-gate was modelled with a neural network, was able to learn the dynamics of hERG activation. This model is purely data-driven, without any predefined mathematical equations, but is still able to capture the dynamics of the ground truth model, slightly better than the candidate model. The NN-d model (green), where a neural network was used to model the discrepancy between the candidate model and the data generating process (the ground truth model), performed similarly to the NN-d model. There is an inherent limitation to modelling the data-generating process dynamics as it requires (at least) two ODEs (hence three states) to fully describe the activation dynamics while we allow only one. However, the neural network models were able to approximate part of the dynamics via the nonlinear mapping between the state variable and its derivative; whereas the candidate model assumes a linear relationship between the state variable and its derivative.

**Figure 4:**
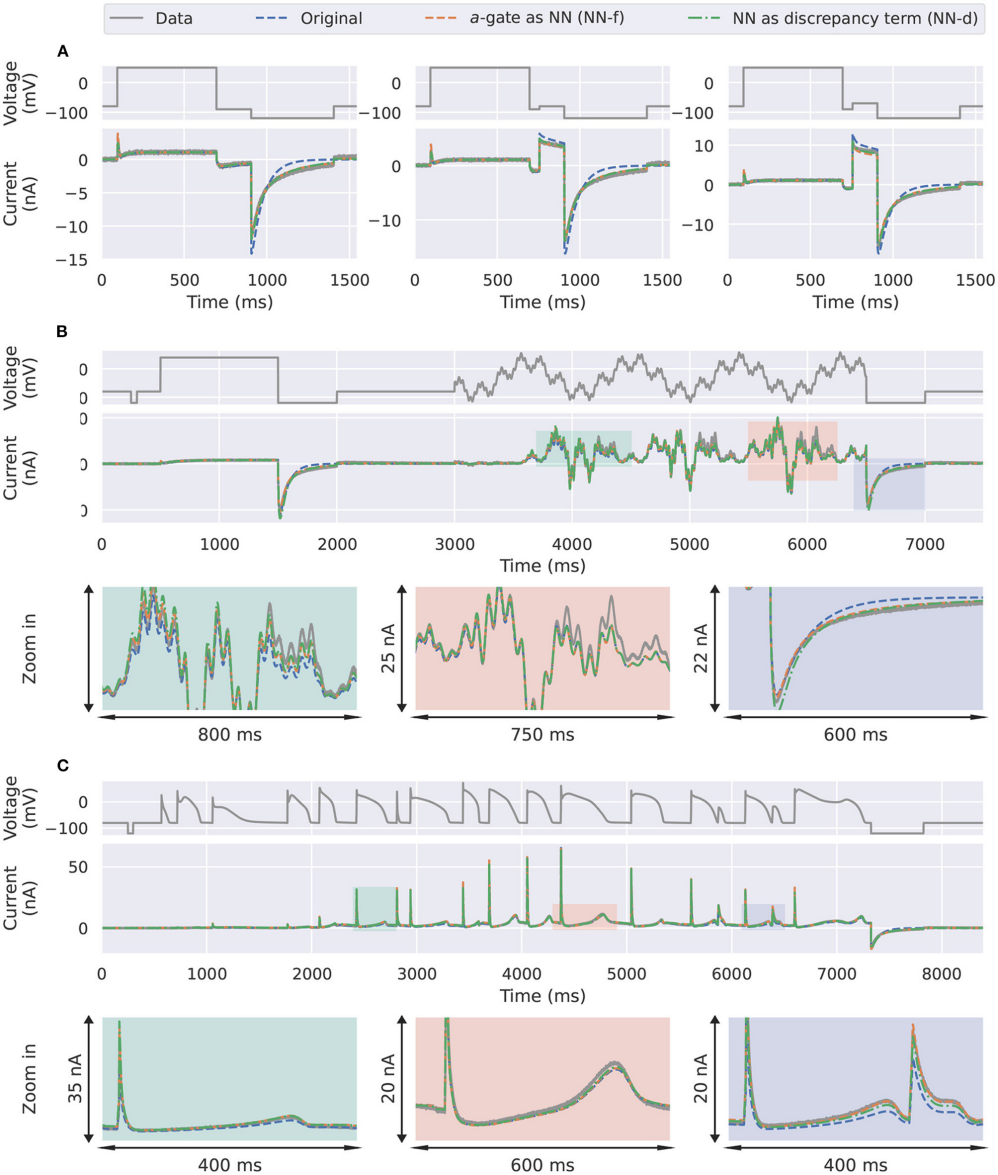
Prediction results for the synthetic data studies. Comparison of the synthetic data generated using the ground truth model (grey) against the candidate *a*-gate model (blue), the *a*-gate modelled using a neural network (NN-f, orange), and the *a*-gate with a neural network discrepancy term (NN-d, green). **(A)** Shows a part of the inactivation protocol (Pr4), showing the first three steps of the protocol. **(B)** Shows the sinusoidal protocol. **(C)** Shows a protocol consists of a series of action potentials. All figures with a blue background are synthetic data examples.

The differences between the three models become even more obvious when it comes to predicting unseen voltage-clamp protocols. Figure 4A shows the first three steps of the inactivation protocol (Pr4) in Beattie et al. (2018). The inactivation *r*-gate is the same for all the models (including the ground truth model); the differences are due to the activation *a*-gates. The ground truth model is equivalent to a model with a second order ODE (Supplementary Material, section S1), see section 4 for more details, whose solution is a sum of two independent exponential functions at constant voltage. Due to the linear relationship between d*a*/d*t* and *a* for the candidate *a*-gate model, by definition the solution *a* for this model can exhibit only a single exponential behaviour at a fixed voltage. Therefore, the candidate model (blue) is incapable of predicting the large “two-time-constant” deactivation current at the end of Pr4. Interestingly, the two neural network models, NN-f (orange) and NN-d (green), were able to predict those deactivation currents quite well, which is thought to be due to the nonlinear d*a*/d*t*-*a* relationship learned by the networks.

For the sinusoidal protocol and the action potential protocol in Figures 4B,C, the two neural network models (orange and green) were able to predict slightly better than the candidate model (blue), which can be seen in the magnifications of the two protocol predictions. For example, a similar deactivation current was elicited at the end of the sinusoidal protocol (the third magnification in Figure 4B, blue); the candidate model gave a single-exponential behaviour whilst the two neural network models closely matched the grey synthetic data generated by the ground truth model. Moreover, there were parts of the sinusoidal protocol and the action potential protocols where the candidate model under-predicted the current, see for example the first magnification in Figure 4B (green) and the last magnification in Figure 4C (blue), whilst the predictions by neural network models were closer to the data. Table 1 shows the mean absolute error of the model simulations (compared against the synthetic data) for each of the protocols (including both the training and prediction results).

**Table 1:** Mean absolute error of the model simulations for the synthetic data study.

	**Training**		**Prediction**		
	**Pr3**	**Pr5**	**Pr4**	**Sinusoidal**	**APs**
Original	0.144	0.166	0.388	0.695	0.463
NN-f	0.113	0.110	0.167	0.453	0.299
NN-d	0.146	0.128	0.165	0.507	0.294

*Comparing the original candidate model, the a-gate modelled using a neural network (NN-f), and the a-gate with a neural network discrepancy term (NN-d) for training results: the activation steady-state protocol (Pr3), and the deactivation time constant protocol (Pr5); and the prediction results: the inactivation protocol (Pr4), the sinusoidal protocol, and the action potential protocol (APs)*.

### 3.2. Applications to CHO Cell Data With Neural Network ODEs

**Figure 5:**
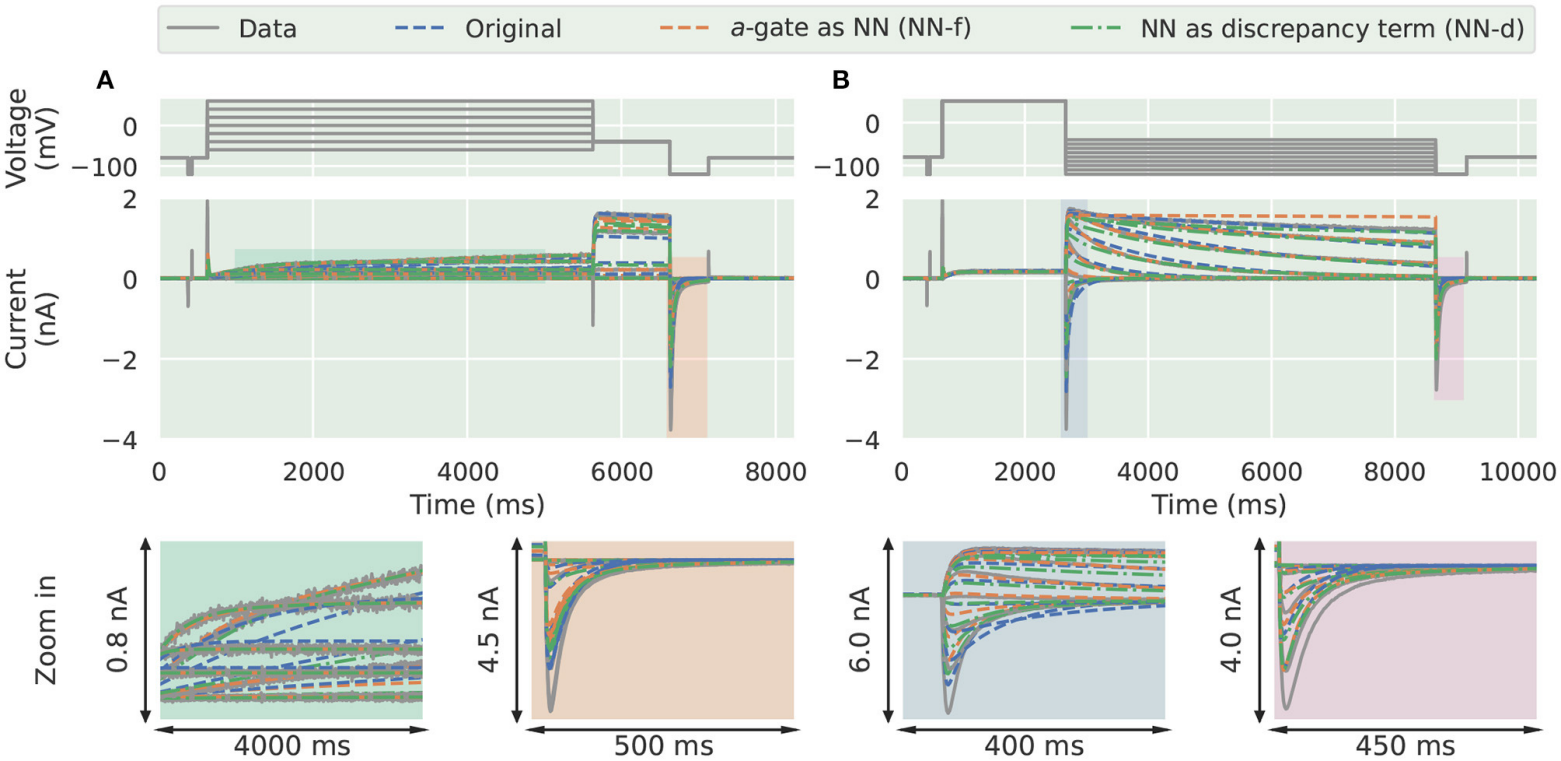
Training results for the experimental data from Beattie et al. (2018). Comparison of the experimental data (grey) against the candidate *a*-gate model (blue), the *a*-gate modelled using a neural network (NN-f, orange), and the *a*-gate with a neural network discrepancy term (NN-d, green). **(A)** Shows the activation steady-state protocol (Pr3) and **(B)** shows the deactivation time constant protocol (Pr5). All figures with a green background are real data examples.

Next we applied the same approach we took in the synthetic data studies to the experimental data collected from a CHO cell overexpressing hERG1a (Beattie et al., 2018). The parameters for the candidate model were adapted from Clerx et al. (2019a, Method 3). The training results with the activation steady-state protocol (Pr3) and the deactivation time constant protocol (Pr5) are shown in Figure 5. The candidate model (blue) failed to capture the transients to the steady state, during the long varying holding steps in Pr3, as shown in the bottom left magnification (green). The two neural network models on the other hand were able to capture such transients to the steady state during the same protocol. A larger magnification to this part of the protocol is shown in Supplementary Figure 5.

**Figure 6:**
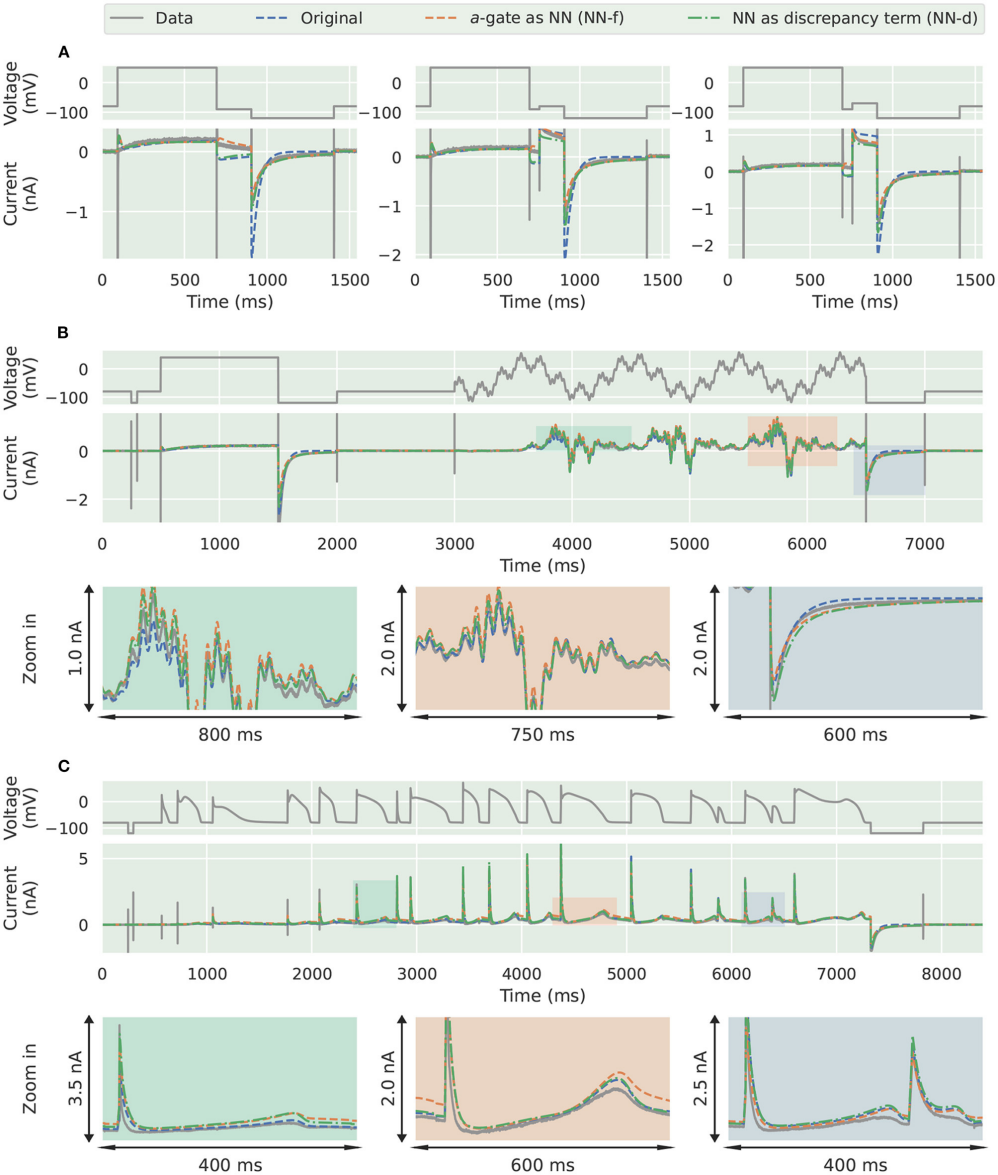
Prediction results for the experimental data from Beattie et al. (2018). Comparison of the experimental data (grey) against the candidate *a*-gate model (blue), the *a*-gate modelled using a neural network (NN-f, orange), and the *a*-gate with a neural network discrepancy term (NN-d, green). **(A)** Shows a part of the inactivation protocol (Pr4), showing the first three steps of the protocol. **(B)** Shows the sinusoidal protocol. **(C)** Shows a protocol consists of a series of action potentials. All figures with a green background are real data examples.

The three trained models were used to predict *unseen* voltage-clamp protocols measured in the same cell during the experiments. Figure 6 shows the prediction results for (Figure 6A) the first three steps of the inactivation protocol, (Figure 6B) the sinusoidal protocol, and (Figure 6C) the action potential wave form protocol. Similarly to the synthetic data studies, the two neural network models were able to better predict the first three steps of the inactivation protocol (Pr4), demonstrating a better description of the deactivation process, as shown in Figure 6A.

However, interestingly the two improved activation models with the neural networks did not show any obvious improvement for the sinusoidal protocol (Figure 6B) and the action potential wave form protocol (Figure 6C); all the three models performed similarly for predicting these two protocols. This could be the fact that the sinusoidal protocol explores the faster dynamics of the hERG current (Beattie et al., 2018), whilst the activation process is rather slow compared to this; similarly for the series of action potential wave forms, as demonstrated in the simulated “phase diagrams” by Clerx et al. (2019a). Therefore, the two neural network models did not show any obvious improvement for these two protocols. Table 2 shows the error of the model simulations for each of the training and prediction protocols.

**Table 2:** Mean absolute error of the model simulations for the CHO cell data.

	**Training**		**Prediction**		
	**Pr3**	**Pr5**	**Pr4**	**Sinusoidal**	**APs**
Original	0.044	0.027	0.066	0.035	0.060
NN-f	0.025	0.025	0.044	0.052	0.107
NN-d	0.029	0.027	0.049	0.035	0.087

*Comparing the original candidate model, the a-gate modelled using a neural network (NN-f), and the a-gate with a neural network discrepancy term (NN-d) for training results: the activation steady-state protocol (Pr3), and the deactivation time constant protocol (Pr5); and the prediction results: the inactivation protocol (Pr4), the sinusoidal protocol, and the action potential protocol (APs)*.

## 4. Discussion

In this paper, we have demonstrated the use of neural networks to model ion channel kinetics. We have shown two approaches for doing this: the first one uses a neural network to fully model the right-hand side of the ODEs; the second one uses a neural network to model only the missing dynamics of the model—discrepancy between a model and the true system. Assessing the model discrepancy in ion channel kinetics is vital to constructing accurate action potential models (Mirams et al., 2016; Clayton et al., 2020; Pathmanathan et al., 2020), but most studies assume correct ion channel kinetics models when fitting maximum conductances of different current types in an action potential model (Kaur et al., 2014; Groenendaal et al., 2015; Johnstone et al., 2016; Lei et al., 2017; Pouranbarani et al., 2019). Previous studies attempted to use different machine learning techniques and statistical methods to model the differences between the mechanistic model and the data. For example, Lei et al. (2020c) used a Gaussian process and autoregressive-moving-average models, respectively, to account for the discrepancy term in ionic currents, the *observables*, i.e., the differences between the solutions of the ODE models and the data. Similarly Creswell et al. (2020) used a flexible noise model to describe the experimental noise, although the residual terms modelled by the flexible noise model were thought to be both correlated noise and model discrepancy. However, given the biophysical justification of the differential equations, we believe the discrepancy is rooted in the mathematical terms of the *right-hand side* of the ODEs, instead of the *solutions* of the ODEs. Therefore, we included the discrepancy term, the neural networks, into the ODEs—neural ODEs.

One of the features of neural networks is their flexibility, which is perhaps both an advantage and a limitation. This flexibility enables neural networks to approximate (almost) any function, making them a powerful universal approximator. However, experimental data are generally imperfect; there are experimental artefacts in the data, for example imperfect series resistance and membrane capacitance compensations, imperfect leak correction, etc., as discussed in Marty and Neher (1995), Sherman et al. (1999), Raba et al. (2013), Lei et al. (2020a,b), and Montnach et al. (2021). Unlike (smaller) biophysical models, with limited flexibility, neural networks might easily absorb such undesired, non-biophysical artefacts into the model, hence making non-physiologically-relevant predictions. It is worth noting that large biophysically-inspired models could also run into the same overfitting issue (Whittaker et al., 2020).

Clerx et al. (2019a) compared the performances of using conventional protocols (such as Pr3, Pr4, and Pr5) and using a condensed protocol (such as the sinusoidal protocol) when fitting an ion channel model. The authors concluded that it was advantageous to use the sinusoidal protocol when fitting the candidate Hodgkin-Huxley model of hERG used in this paper (Figure 1). The biggest differences between the neural network models and the candidate model are the predefined model structure and the number of degrees of freedom. Some of the condensed protocols, such as the sinusoidal protocol in Beattie et al. (2018) and the “staircase” protocol in Lei et al. (2019a,b), were designed to explore the dynamics of a given model rapidly. However, in this case, given the lack of model structure for the neural network models, these condensed protocol designs may not be the most appropriate choices. When training neural ODEs, it has been suggested to use large numbers of short time series data (Chen et al., 2018; Zhong et al., 2020; Su et al., 2021); however, it is often not practical to collect large numbers of short time series by restarting the voltage-clamp experiments, as it would require bringing the cell to steady state many times in order to obtain reliable initial conditions for solving the differential equations. The central idea of using multiple shorter time series data is to explore different regions of dynamics for the system to be modelled (Wu and Xiu, 2019; Su et al., 2021), which is the same as exploring the state space in our approach. We therefore decided to choose training protocols that cover the state space as much as possible; this also ensures the trained neural network models do not *extrapolate*—make predictions outside the training space (see later for a demonstration of such a pitfall). Supplementary Figure 6 shows the state space covered by the sinusoidal protocol, which is not as wide as those shown in Figure 2. When training neural ODE models it may therefore be more suitable to use protocols that cover the possible input space as widely as possible—here a combination of Pr3 and Pr5 for hERG activation appears to do this well.

In this paper, we have proposed a novel way of estimating the dynamics of the ion channel model, termed “state space estimation.” The underpinning of the proposed method is similar to some methods suggested in the literature for training neural ODEs. For example, Su et al. (2021) suggested using pairs of state variables at two adjacent time instants as the training data for the neural networks, where their neural network structure is a variant of residual networks. They were effectively approximating the derivatives using the first-order forward finite difference method with a fixed time step, although this would greatly amplify any noise present in the data (Chartrand, 2011). We have relaxed this limitation by allowing variable time steps and have estimated the derivatives using splines, one could also use different methods for estimating the derivatives under our framework (such as Chartrand, 2011; Van Breugel et al., 2020). Su et al. (2021) also assumed one could independently observe all the gating variables, which is not feasible in standard electrophysiology experiments that record only the total current.

Another approach for training neural ODEs is the adjoint method suggested by Chen et al. (2018), which back-propagates the derivatives of the neural network parameters from the solutions for constant memory cost. Such a method is an attractive alternative to our method, however when modelling typically long and dense time series data from voltage-clamp experiments, training neural networks using backpropagation through the ODE solutions is extremely slow. Our method provides a computational speed up at a low memory cost, which makes it even possible to train on CPUs.

Neural networks are excellent as a universal approximation mechanism, but they are *not* a reliable function extrapolation mechanism (Haley and Soloway, 1992; Chapter 6 of Livshin, 2019). That means these neural networks are excellent in predicting the approximated function values *within* the training space. However, they are not suitable for predicting the function values *outside* the training space. To demonstrate this issue, here we attempt to deliberately use a combination of the activation steady state protocol (Pr3) and the inactivation protocol (Pr4), which were not designed to thoroughly probe the activation of hERG, to train our NN-f model.

**Figure 7:**
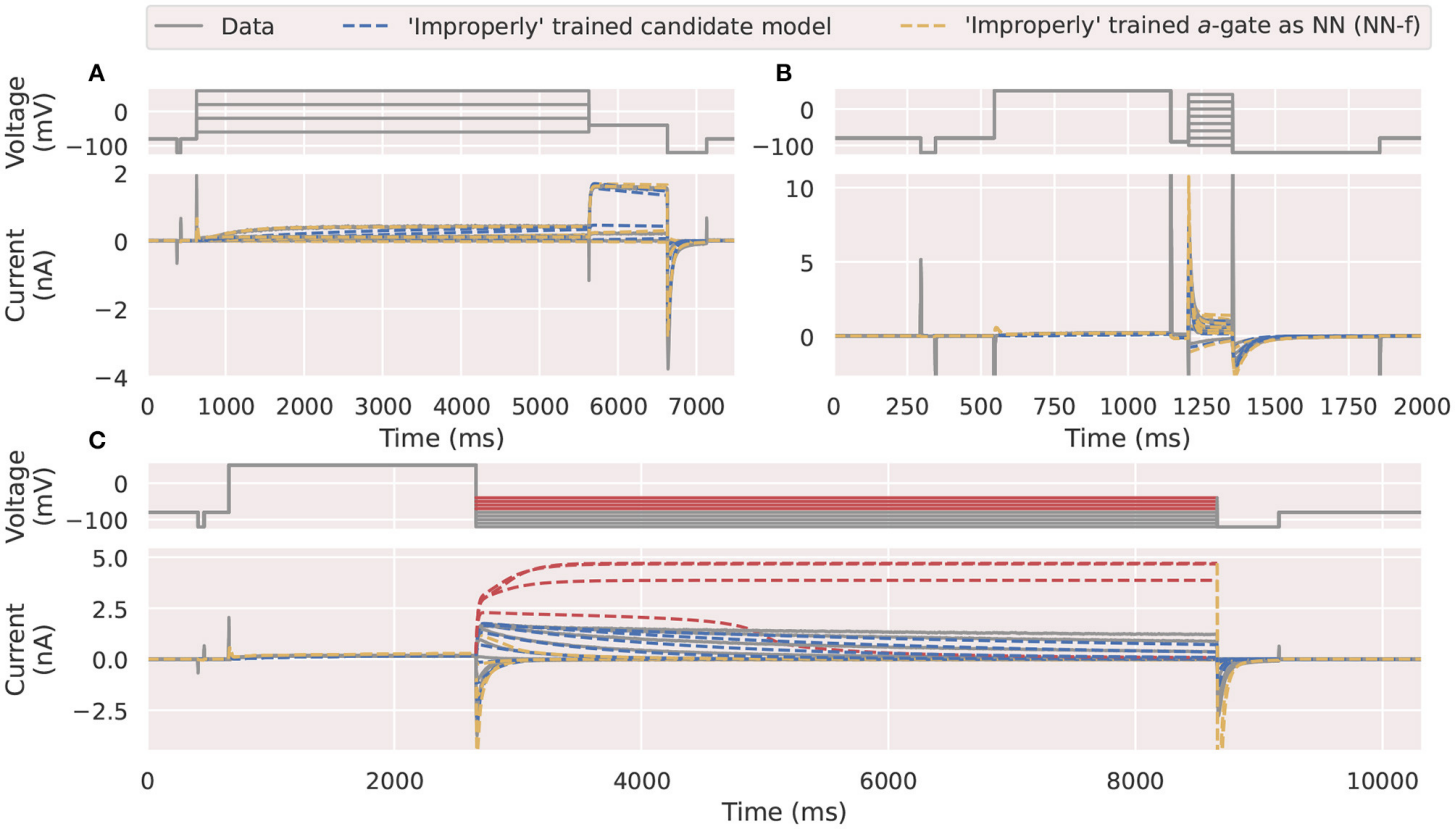
An example of neural ODE performance using an inappropriate choice of training protocols. Comparison of the experimental data (grey) against the *a*-gate modelled using an “incorrectly” trained candidate model (blue) and neural network (NN-f, orange). The neural network was trained using **(A)** the activation steady state protocol (Pr3) and **(B)** the inactivation protocol (Pr4), where only parts of the protocols are shown for visualization purpose. **(C)** Shows the mechanistic candidate model makes reasonable predictions (blue) for this deactivation time constant protocol (Pr5) but the NN-f model failed to predict accurately, with four of the currents under higher test voltages (-70 to -40 mV) highlighted in red. All figures with red backgrounds are trained on Pr3/Pr4.

**Figure 8:**
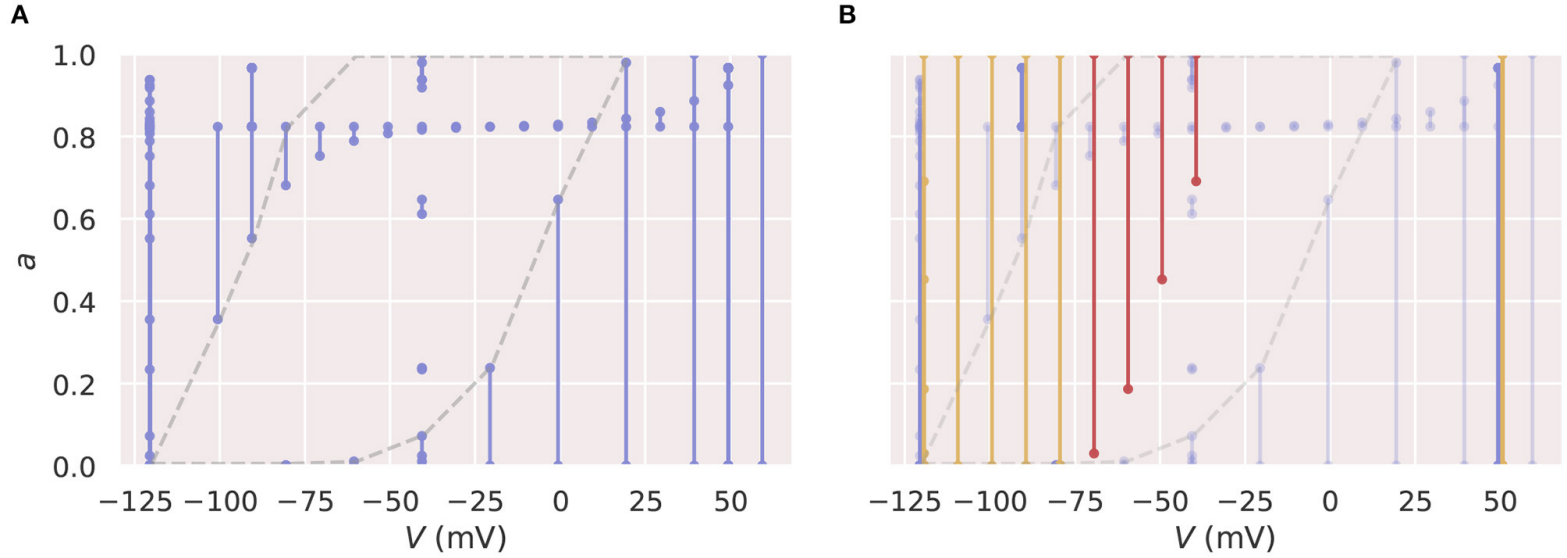
Two-dimensional state spaces illustrating the inappropriate training protocol for a neural ODE. The lines on these diagrams indicate states occupied at some point in time in simulations using the candidate model, with a dot for the state at the start and end of each voltage step. **(A)** Shows the state space spanned by the Pr3 and Pr4 training protocols (blue). The grey dashed line highlights a large region of very sparse training data. **(B)** The same state space with trajectories required by the prediction protocol, Pr5, highlighted (orange and red). The sections highlighted in red in Figure 7 with very bad predictions are also shown in red here. It is evident that the neural ODE makes “bad” predictions when extrapolating into the centre of the sparse region of training samples. All figures with red backgrounds are trained on Pr3/Pr4.

Figure 7 shows the training (Figures 7A,B) and prediction (Figure 7C) results, where the “badly trained” NN-f model failed to predict the parts of the deactivation protocol (Pr5) that are highlighted in red, whilst still performing very well with the training protocols. To illustrate the probable cause, Figure 8A shows a two-dimensional state space explored by the training protocols (see also Supplementary Figure 7). We see that there is a large “unexplored” region in the training protocols Pr3/4. This region is used for predictions of Pr5, and the worst predictions (indicated in red in Figures 7C, 8B) are those toward the centre of the “unexplored” region. This cautionary example suggests that Pr3/4 would be inappropriate training for a neural ODE and it is particularly important that we choose appropriate voltage-clamp protocols when training a neural ODE model. That is, we believe the training space should cover the full dynamics of interest within the state space, such that when we use the model to perform “predictions,” we are predicting a different state space trajectory within, or very close to, the trained state space. Note that Figure 7 also shows that a mechanistic model (candidate model, blue) fitted to Pr3 and Pr4 would give “reasonable” predictions for Pr5, although not as good as those in Figure 5 (see Clerx et al., 2019a). This performance is thought to be due to the mechanistic equations appropriately restricting model predictions—resulting in far more reliable and biophysically-based *extrapolation*.

In this paper we have used voltage-gated ion channels as an example, one could also generalise the neural network model to include other external effects or control variables in a similar fashion as we demonstrated with the membrane voltage *V* in voltage-clamp experiments. We can write the neural network models in Equations (2) and (9) as

(17) $$\begin{array}{l} \frac{\text{d}x}{\text{d}t}=N\left(x,u\right) \end{array}$$

and

(18) $$\begin{array}{l} \frac{\text{d}x}{\text{d}t}=f+N\left(x,u\right), \end{array}$$

which explicitly includes an external control variable ***u***. These external effects could be for example compound concentration, energy source (e.g., ATP concentration for pumps), luminance levels for light-gated ion channels, etc. However, the drawback of including more (input/control) variables to the model is that we have to train a model in higher dimensions (see discussion below).

The proposed ways of embedding a neural network into the ODEs, NN-f, and NN-d, are two of many possible ways of structuring the neural network models. For example Zhong et al. (2020) and Yazdani et al. (2020) suggested *replacing* only *part of* an ODE system with a neural network. For Hodgkin-Huxley or Markov models, a way of doing this would be to relax the rate assumptions. That is, instead of using an exponential form to model the transition rates α(*V*) and β(*V*), we could model them with a neural network such that Equation (3) becomes

(19) $$\begin{array}{l} f\left(x,V\right)=N_{\alpha }\left(V\right)\left(1-x\right)-N_{\beta }\left(V\right)x. \end{array}$$

N_α_ and N_β_ are the outputs of a neural network N with an input *V*. This form indeed imposes good mechanistic structure, and is easier to interpret and train compared to the two proposed neural network models in this work; this particular form implicitly defines the bounds for the solution *x* to be [0, 1], making *x* can still be interpreted as the open probability. However, depending on the form of discrepancy, Equation (19) may not be flexible enough to model the missing dynamics. It implicitly assumes that the rate of the state d*x*/d*t* is linear in the state *x*, which is not suitable to correct the differences shown in Figure 3 (two time constants of deactivation) as our methods did.

In theory, we can even model the gating dynamics using *higher order* ODEs. For example, a second order ODE in general can be written as

(20) $$\begin{array}{l} \frac{{\text{d}}^{2}x}{\text{d}t^{2}}=N\left(V,x,\frac{\text{d}x}{\text{d}t}\right). \end{array}$$

This type of second order ODE can be solved as a system of first order ODEs by considering it as

(21) $$\begin{array}{l} \frac{\text{d}v}{\text{d}t}=N\left(V,x,v\right) \end{array}$$

(22) $$\begin{array}{l} \frac{\text{d}x}{\text{d}t}=v, \end{array}$$

which is a type of augmented neural differential equation (Norcliffe et al., 2020). Such a model is equivalent to a generalised three-state Markov model with one open state (Supplementary Material, section S1 shows how to rewrite a three-state model into a second order ODE, where its right-hand side is replaced by a neural network in a similar fashion to the NN-f model). In general, to model an *n*^*th*^ order ODE, we could have a neural ODE of the form

(23) $$\begin{array}{l} \frac{{\text{d}}^{n}x}{\text{d}t^{n}}=N\left(V,x,\frac{\text{d}x}{\text{d}t},\ldots ,\frac{{\text{d}}^{n-1}x}{\text{d}t^{n-1}}\right). \end{array}$$

We therefore run back into a *model selection* challenge, which is one of the main challenges within conventional ion channel modelling—which model is the most suitable one to use—except we need to select the model in terms of the order of the neural ODEs and how to best include the neural network in the ODEs. Another challenge is that the higher the order, the higher the state space dimension (for an (*n* + 1)^*th*^ order ODE, we have (*n* + 2)-dimensions: *V*, *x*, d*x*/d*t*, …, d^*n*^*x*/d*t*^*n*^) and the harder it is to train a neural network. With the concept of covering the state space for training the dynamics, we are faced with the curse of dimensionality as we go to higher orders, because it is practically impossible to collect training data that cover a large proportion of the hyper-volume within the state space in high dimensions. Also, neural ODE models of this form do not impose bounds to the solutions in general, and predictions for probabilities by these models could go outside [0, 1] during extrapolation.

On the note of model selection, Menon et al. (2009) attempted to theoretically optimise model structure in addition to the rate parameters through a genetic algorithm; Mangold et al. (2021) suggested a systematic way of proposing a set of Markov models by treating Markov structures as different graphs. Both approaches try to deal with a large scale of model selection; in particular Mangold et al. (2021) showed that there are more than 10^8^ unique graphs (Markov model structures) even for only ten-state models. The number of possible unique graphs *combinatorially* explodes as the number of states increases, although a benefit of exploring different Markov structures is that the obtained best model has a potentially-explainable biophysical structure. On the other hand, for up to 10-state models, neural ODEs would, in theory, simplify the model selection problem from > 10^8^ models to 10 models—by selecting the correct order of the ODE, although we anticipate a neural network model with nine hidden states would be extremely difficult to train accurately. This simplification is achieved by absorbing the selection of all the possible unique graphs for a given number of states (the order of the ODEs) into a single optimisation problem (i.e., training the neural network weights). Moreover, using neural networks to model the right-hand side of the ODE could allow some out-of-formalism behaviour (e.g., Lowen et al., 1999)—if the real channels are doing anything more exotic than the models assume. Although we see great potential in using neural ODE modelling approaches that we demonstrated in this paper for ion channel modelling, we believe this approach is still in its infancy; there are several limitations that we have to overcome before these neural ODE models can be used as confidently as the standard ion channel models.

## 5. Conclusion

In this paper, we have developed a method for training neural ODEs for ion channel models. We assessed the performance of neural ODEs with synthetic data studies and applied them to experimental data for hERG. We found that the neural ODEs were able to recover some of the missing dynamics in the synthetic data studies, whilst they were not particularly outperforming a standard Hodgkin and Huxley-style model used in the literature when applied to experimental data. Neural ODE modelling approach has great potential for handling model discrepancy or misspecification, although currently it still has strong limitations in terms of reliable extrapolation and training for higher order ODEs.

## Data Availability Statement

Publicly available datasets were analysed in this study. This data can be found at: https://github.com/chonlei/neural-ode-ion-channels and is archived on Zenodo at https://doi.org/10.5281/zenodo.5095091.

## Author Contributions

CLL and GM designed and conceptualised the research and wrote and approved the final version of the manuscript. CLL wrote simulation codes, performed the analysis, and generated the results figures. Both authors contributed to the article and approved the submitted version.

## Conflict of Interest

The authors declare that the research was conducted in the absence of any commercial or financial relationships that could be construed as a potential conflict of interest.

## Publisher's Note

All claims expressed in this article are solely those of the authors and do not necessarily represent those of their affiliated organizations, or those of the publisher, the editors and the reviewers. Any product that may be evaluated in this article, or claim that may be made by its manufacturer, is not guaranteed or endorsed by the publisher.
