# Supplementary material for: Neural Network Differential Equations For Ion Channel Modelling
Source: Front Physiol. 2021 Aug 4;12:708944. doi: 10.3389/fphys.2021.708944 (PMC8371386; doi:10.3389/fphys.2021.708944)
Supplement: Supplementary file 1 [file Data_Sheet_1.PDF]

# Supplementary Material: Neural network differential equations for ion channel modelling


***Chon Lok Lei*** [1,2,3,*] ***and Gary R. Mirams*** [4]

*[1] Institute of Translational Medicine, Faculty of Health Sciences, University of Macau, Macau, China, [2] Department of Biomedical Sciences, Faculty of Health Sciences, University of Macau, Macau, China, [3] School of Mathematical Sciences, Faculty of Science and Engineering, University of Nottingham, Ningbo, China, [4] Centre for Mathematical Medicine & Biology, School of Mathematical Sciences, University of Nottingham, Nottingham, United Kingdom*

[*] ***Correspondence:*** *Chon Lok Lei, chonloklei@um.edu.mo*


## S1 RE-WRITING THE THREE-STATE MODEL

*The three-state activation model for generating the synthetic data in the main text (Figure 1 'ground truth') can be written as a second order differential equation. The three-state model is given by:*

$$\frac{\mathrm{d}O}{\mathrm{d}t} = k_2 C_1 - k_1 O, \tag{S1}$$

$$\frac{\mathrm{d}C_1}{\mathrm{d}t} = k_1 O - k_2 C_1 - k_3 C_1 + k_4 C_2, \tag{S2}$$

$$C_2 = 1 - O - C_1, \tag{S3}$$

*where $O$ is the open state, $C_1, C_2$ are the two closed states. To simplify the notation, the rate constants in the diagram are renamed as follows: $k_1 = \beta_a$, $k_2 = \alpha_a$, $k_3 = \beta'_a$, and $k_4 = \alpha'_a$. By taking a full derivative of Eq. (S1) and substituting Eqs. (S2) and (S3) to it, we have*

$$\frac{\mathrm{d}^2 O}{\mathrm{d}t^2} = -\left(k_1 + k_2 + k_3 + k_4\right) \frac{\mathrm{d}O}{\mathrm{d}t} - \left(k_1 k_3 + k_1 k_4 + k_2 k_4\right) O + k_2 k_4. \tag{S4}$$

*Alternatively it can expressed as*

$$\frac{\mathrm{d}^2 O}{\mathrm{d}t^2} = \kappa_1 \frac{\mathrm{d}O}{\mathrm{d}t} + \kappa_2 O + \kappa_3, \tag{S5}$$

*where $\kappa_1 = -(k_1 + k_2 + k_3 + k_4)$, $\kappa_2 = -(k_1 k_3 + k_1 k_4 + k_2 k_4)$, and $\kappa_3 = k_2 k_4$.*

# S2 SUPPLEMENTARY TABLES

| | 10 nodes | 100 nodes | 200 nodes | 500 nodes |
|---|---|---|---|---|
| 1 layer | $s_{1,1}$ | $s_{1,2}$ | $s_{1,3}$ | $s_{1,4}$ |
| 5 layers | $s_{2,1}$ | $s_{2,2}$ | $s_{2,3}$ | $s_{2,4}$ |
| 10 layers | $s_{3,1}$ | $s_{3,2}$ | $s_{3,3}$ | $s_{3,4}$ |

| | Training | | | Prediction | | | |
|---|---|---|---|---|---|---|---|
| | Pr3 | Pr5 | Mean | Pr4 | Sinusoidal | APs | Mean |
| $s_{1,1}$ | 0.081 | 0.051 | 0.066 | 0.057 | 0.040 | 0.074 | 0.057 |
| $s_{1,2}$ | 0.065 | 0.022 | 0.044 | 0.045 | 0.051 | 0.114 | 0.070 |
| $s_{1,3}$ | 0.035 | 0.028 | 0.032 | 0.044 | 0.055 | 0.112 | 0.070 |
| $s_{1,4}$ | 0.802 | 0.026 | 0.414 | 0.045 | 0.051 | 0.113 | 0.070 |
| $s_{2,1}$ | 0.024 | 0.081 | 0.053 | 0.049 | 0.051 | 0.121 | 0.074 |
| $s_{2,2}$ | 0.040 | 0.027 | 0.034 | 0.044 | 0.047 | 0.105 | 0.065 |
| $s_{2,3}$ | 0.025 | 0.025 | 0.025 | 0.044 | 0.052 | 0.107 | 0.067 |
| $s_{2,4}$ | 0.034 | 0.027 | 0.031 | 0.045 | 0.053 | 0.117 | 0.072 |
| $s_{3,1}$ | 0.026 | 0.067 | 0.047 | 0.048 | 0.051 | 0.118 | 0.072 |
| $s_{3,2}$ | 0.146 | 0.023 | 0.085 | 0.045 | 0.055 | 0.104 | 0.068 |
| $s_{3,3}$ | 0.028 | 0.026 | 0.027 | 0.044 | 0.048 | 0.111 | 0.068 |
| $s_{3,4}$ | 0.166 | 0.032 | 0.099 | 0.045 | 0.046 | 0.101 | 0.064 |

**Table S1. Grid search for the hyperparameters of the neural network ODE.** It shows the mean absolute error of the NN-f model when applied to the CHO cell data.

| | $\alpha_a$ | $\beta_a$ | $\alpha'_a$ | $\beta'_a$ | $\alpha_r$ | $\beta_r$ |
|---|---|---|---|---|---|---|
| $A$ ($\mathrm{ms}^{-1}$) | $5.95 \times 10^{-5}$ | $4.76 \times 10^{-3}$ | 0.207 | $1.26 \times 10^{-3}$ | $8.01 \times 10^{-3}$ | 0.0962 |
| $B$ ($\mathrm{mV}^{-1}$) | 0.121 | $-3.49 \times 10^{-6}$ | 0.0331 | $-0.0225$ | $-0.0244$ | 0.0226 |

**Table S2. The ground-truth model kinetic parameters used for generating the synthetic data.** All the $A$ parameters have units of $\mathrm{ms}^{-1}$ and all the $B$ parameters have units of $\mathrm{mV}^{-1}$.

| | $\alpha_a$ | $\beta_a$ | $\alpha_r$ | $\beta_r$ |
|---|---|---|---|---|
| $A$ ($\mathrm{ms}^{-1}$) | $5.69 \times 10^{-5}$ | $3.52 \times 10^{-5}$ | $8.01 \times 10^{-3}$ | 0.0962 |
| $B$ ($\mathrm{mV}^{-1}$) | 0.117 | $-4.97 \times 10^{-2}$ | $-0.0244$ | 0.0226 |

**Table S3. The fitted kinetic parameters of the candidate model in the synthetic data.** All the $A$ parameters have units of $\mathrm{ms}^{-1}$ and all the $B$ parameters have units of $\mathrm{mV}^{-1}$.

## S3 SUPPLEMENTARY FIGURES

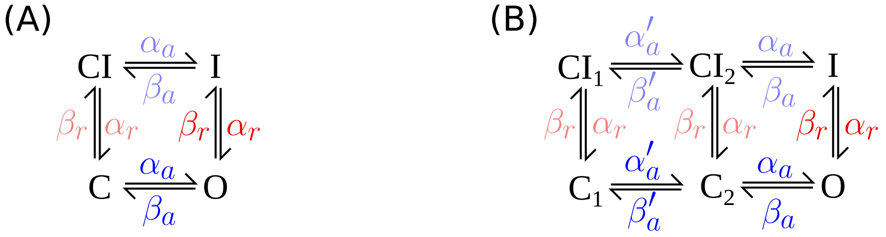


**Figure S1. Markov structure diagrams for the models of hERG used in synthetic data studies.** Both models are Hodgkin-Huxley style for independent inactivation due to the symmetry in the rate constants. **(A)** Shows the candidate model. **(B)** Shows the ground truth model that generates the synthetic data.

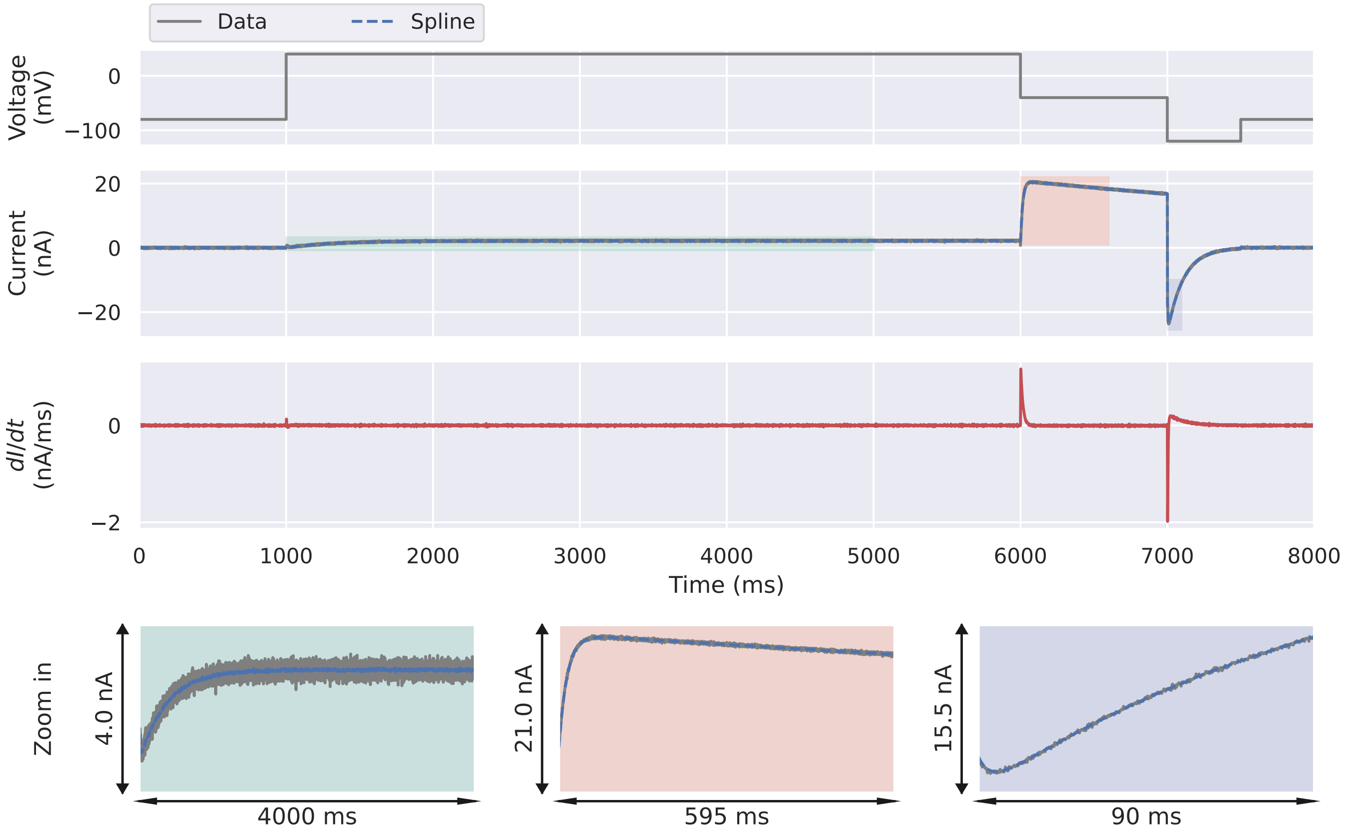


**Figure S2. An example of spline fitting with the synthetic data.** The training data generated using the candidate model (grey) are compared against the fitted spline curves (blue). The top panel shows the voltage-clamp protocols, the second panel shows the currents, the third panel shows the estimated derivative of the current using the spline, and the bottom panels shows the magnification of part of the currents.

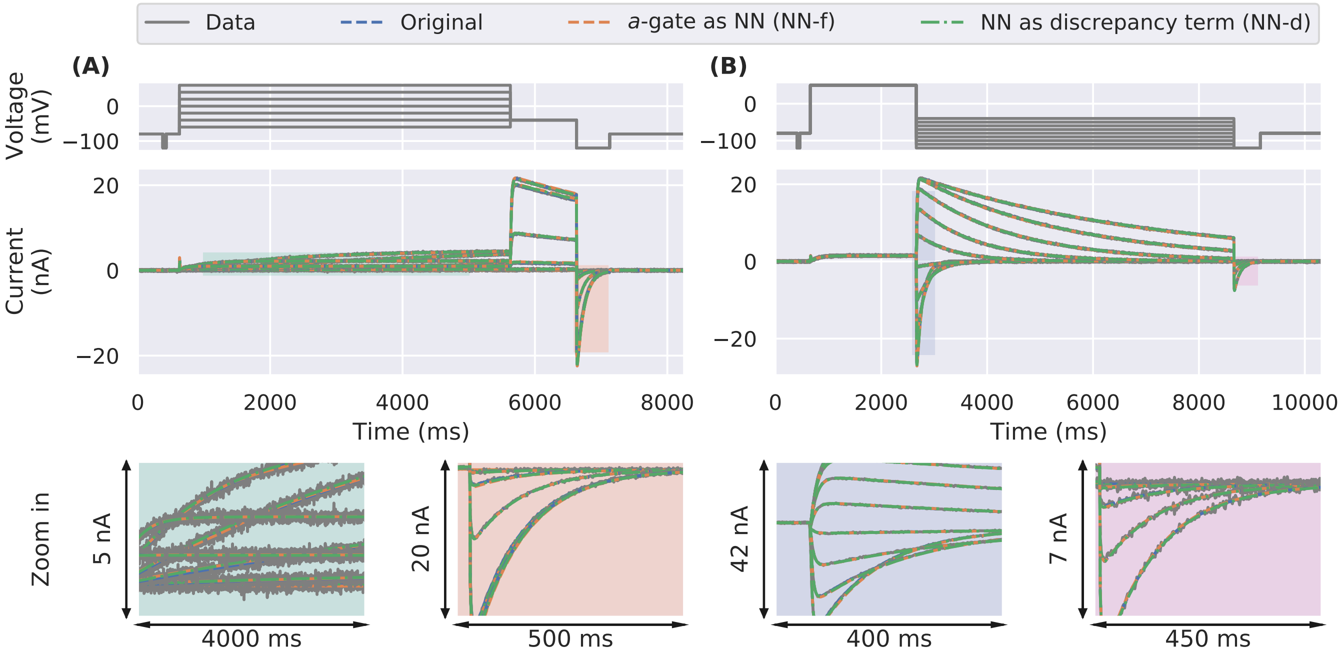


**Figure S3. Training results for the candidate model synthetic data studies.** Comparison of the training data generated using the candidate model (grey) against the no-noise candidate model (blue), the $a$-gate modelled using a neural network (NN-f, orange), and the $a$-gate with a neural network discrepancy term (NN-d, green). **(A)** Shows the activation steady-state protocol (Pr3) and **(B)** shows the deactivation time constant protocol (Pr5). The top panel shows the voltage-clamp protocols, the middle panel shows the currents, and the bottom panels shows the magnification of part of the currents.

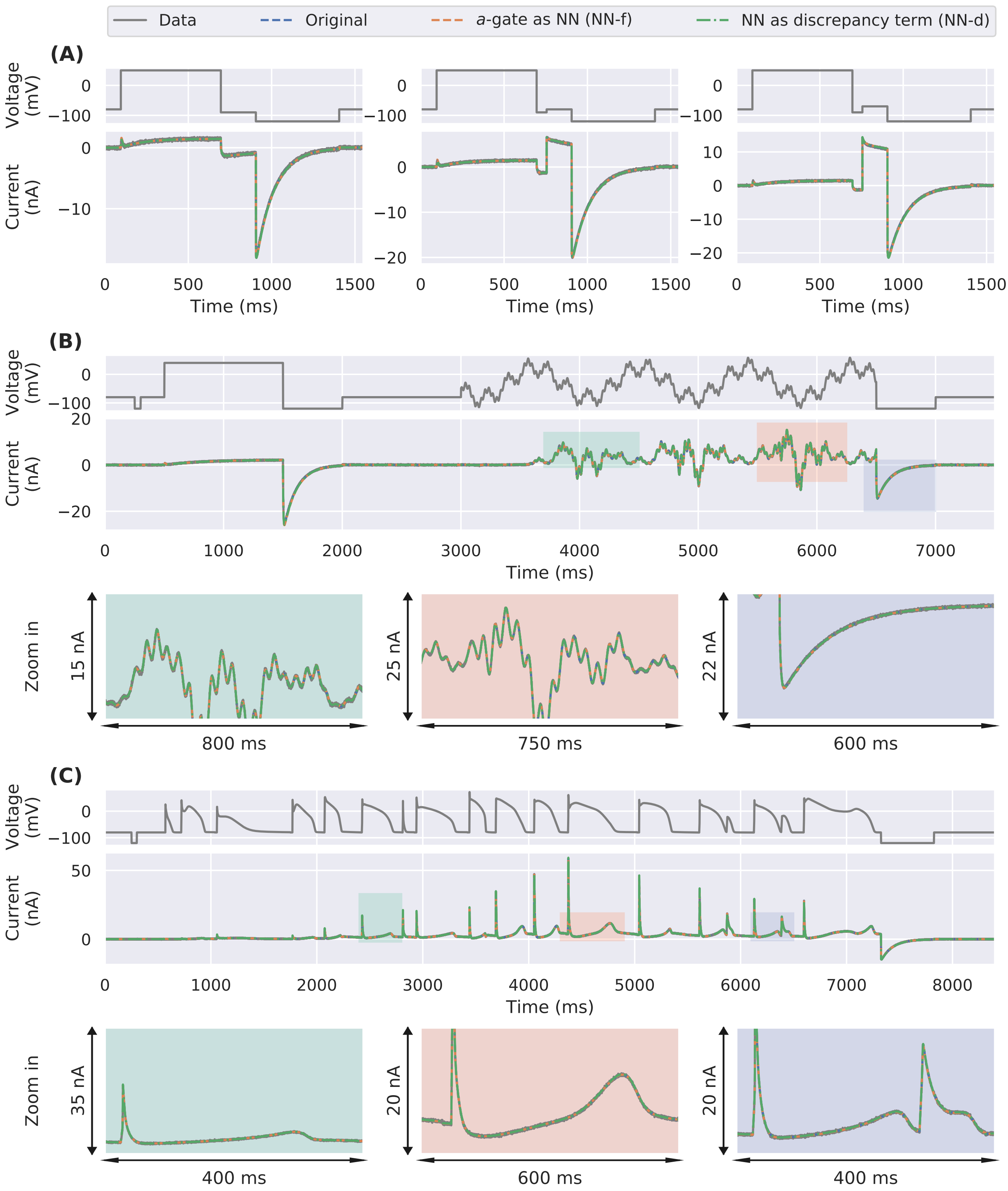


**Figure S4. Prediction results for the candidate model synthetic data studies.** Comparison of the training data generated using the candidate model (grey) against the no-noise candidate model (blue), the $a$-gate modelled using a neural network (NN-f, orange), and the $a$-gate with a neural network discrepancy term (NN-d, green). **(A)** Shows a part of the inactivation protocol (Pr4), showing the first three steps of the protocol. **(B)** Shows the sinusoidal protocol. **(C)** Shows a protocol consists of a series of action potentials.

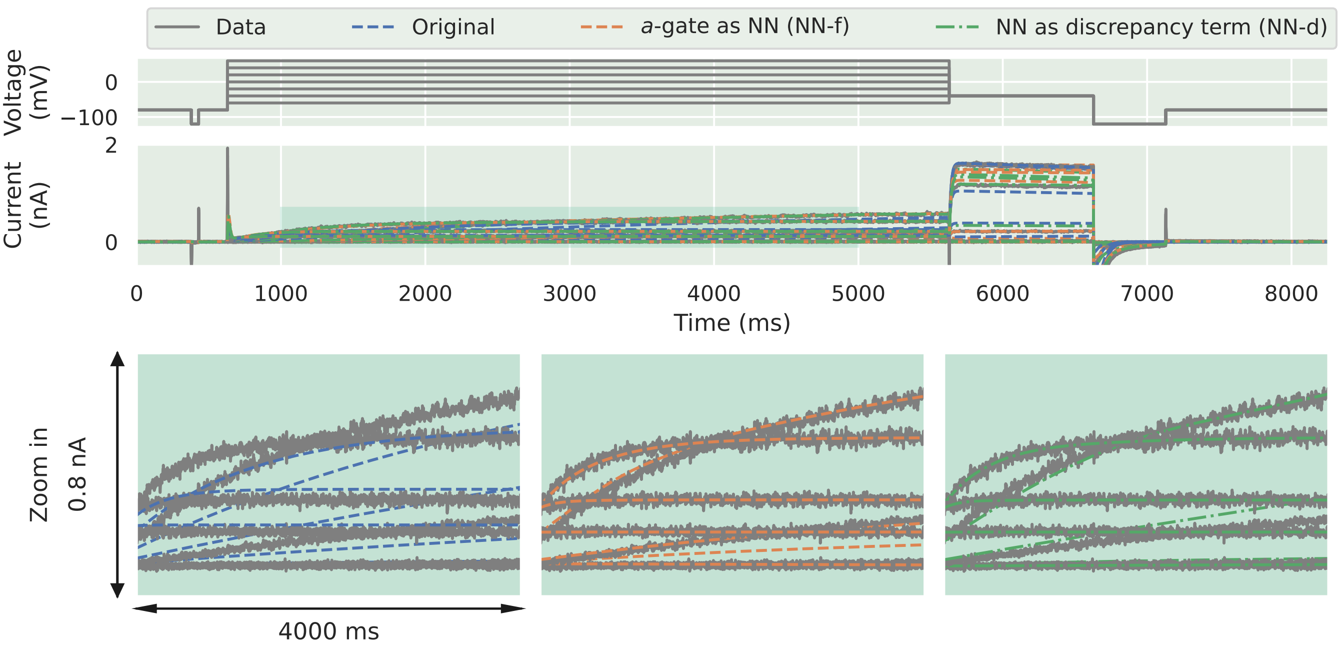


**Figure S5. Training results for the CHO cell data.** Comparison of the experimental data (grey) recorded under the activation steady-state protocol (Pr3) against the candidate model (blue), the $a$-gate modelled using a neural network (NN-f, orange), and the $a$-gate with a neural network discrepancy term (NN-d, green).

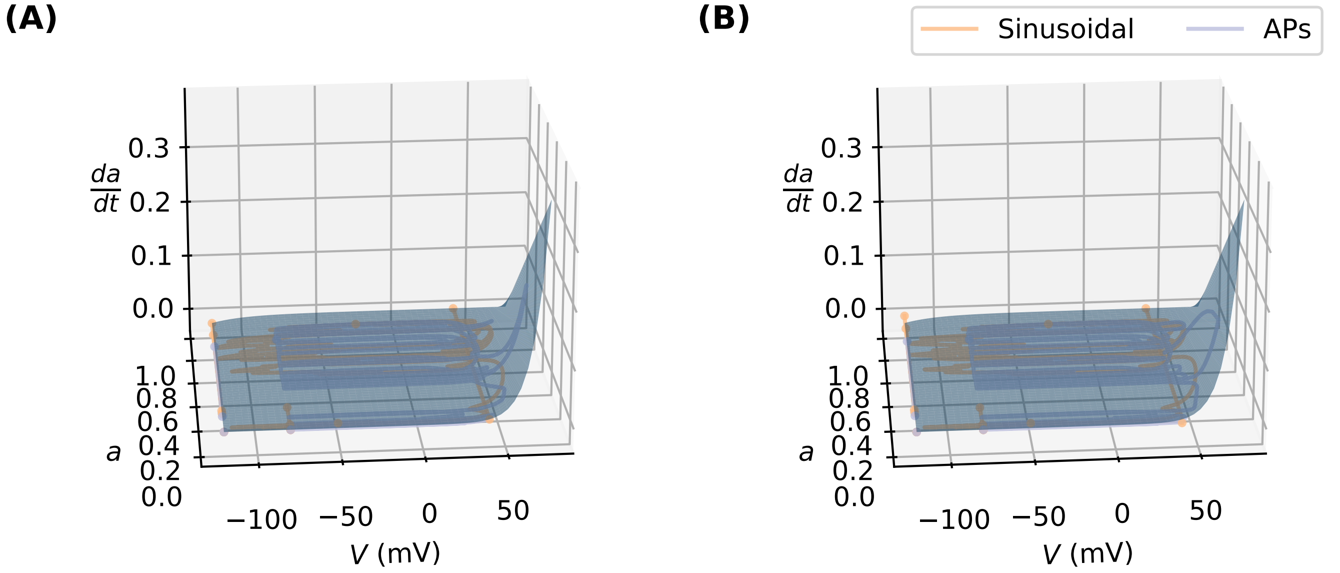


**Figure S6. An example of the phase space simulated in synthetic data studies.** The phase space of the candidate model (blue surface) is shown as blue surfaces. The simulated sinusoidal protocol (orange lines) and the simulated action potential series protocol (APs, purple lines) are shown for **(A)** the candidate model and **(B)** the ground truth model. Each dot at the two ends of the lines indicates a voltage step jump in the protocols.

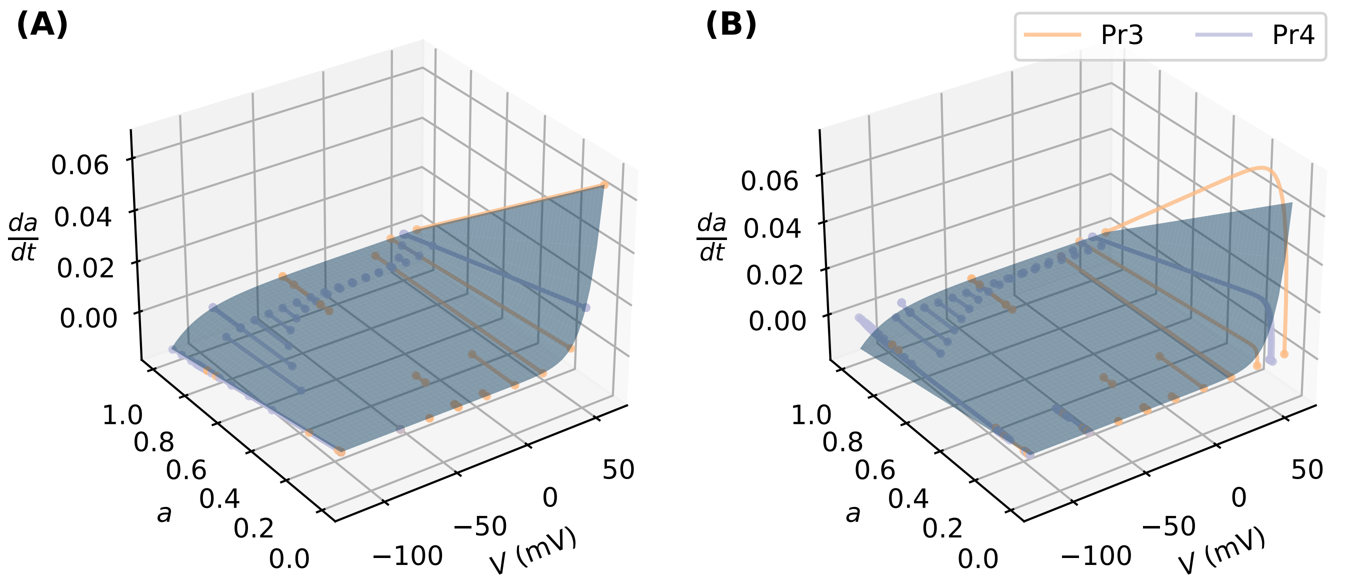


**Figure S7. An example of the phase space simulated in synthetic data studies.** The phase space of the candidate model (blue surface) is shown as blue surfaces. The simulated activation steady-state protocol (Pr3, orange lines) and the simulated inactivation time constant protocol (Pr4, purple lines) are shown for **(A)** the candidate model and **(B)** the ground truth model. Each dot at the two ends of the lines indicates a voltage step jump in the protocols.
